# Proof of Concept: Effects of an Immune-Enhancing Formula on Clinical Markers of Critical Coronavirus Disease 2019 Cases

**DOI:** 10.3390/biomedicines13020309

**Published:** 2025-01-27

**Authors:** Yi-Cheng Hou, Su-Ting Lin, Chin-Hsuan Yang, Kuo-Wang Tsai, Jing-Huei Wu, Hsiang-Yu Huang, Wen-Lin Su

**Affiliations:** 1 Department of Nutrition, Taipei Tzu Chi Hospital, Buddhist Tzu Chi Medical Foundation, New Taipei City 231, Taiwan; anny.houyicheng@gmail.com (Y.-C.H.); shuting861001@gmail.com (S.-T.L.); chin.hsuan.ee@gmail.com (C.-H.Y.); rita@tzuchi.com.tw (J.-H.W.); 2 Department of Medical Research, Taipei Tzu Chi Hospital, Buddhist Tzu Chi Medical Foundation, New Taipei City 231, Taiwan; kwtsai6733@gmail.com; 3 Department of Respiratory Therapy, Taipei Tzu Chi Hospital, Buddhist Tzu Chi Medical Foundation, New Taipei City 231, Taiwan; xd510131@tzuchi.com.tw; 4 Division of Pulmonary Medicine, Taipei Tzu Chi Hospital, Buddhist Tzu Chi Medical Foundation, New Taipei City 231, Taiwan; 5 School of Medicine, Tzu-Chi University, Hualien 970, Taiwan

**Keywords:** COVID-19, enteral nutrition, food, specialized, APACHE, IL-10

## Abstract

**Background/Objectives**: The rapid viral spread observed in coronavirus disease 2019 (COVID-19) is capable of inducing the secretion of excessive inflammatory cytokines. The resulting multi-organ damage is a severe complication that can be attenuated through adequate nutrition. Formulae enhanced with either glutamine or arginine are conditionally essential amino acids that have been proven to improve the condition of hospitalized patients. This retrospective study aimed to investigate the effects of administering an immune-enhancing enteral formula enhanced with arginine and glutamine on the clinical signs and biomarkers of patients with severe COVID-19. **Methods:** After checking the data of 232 patients enrolled in the biobank for completeness and eligibility, 31 patients with severe COVID-19 in the intensive care unit at Taipei Tzu Chi Hospital were grouped based on the type of enteral formula used: 16 patients received the control formula, and 15 patients received the immune-enhancing formula. Baseline characteristics, clinical signs, and inflammatory markers were analyzed for differences. **Results:** An increase in IL-10 levels in the intervention group was observed (*p* = 0.048). Changes in other inflammatory cytokine levels were insignificant. **Conclusions:** Providing an enteral formula enriched with glutamine and arginine to severe COVID-19 patients may help improve their anti-inflammatory marker levels. Further interventional study utilizing enteral formula enriched with glutamine and arginine is needed to confirm the findings of this study.

## 1. Introduction

Coronavirus disease 2019 (COVID-19) has emerged as a novel epidemic over the past four years. As of 2 May 2020, there were 3 million confirmed cases worldwide, with nearly 230,000 fatalities attributed to COVID-19. Moderate to severe cases of COVID-19 are often accompanied by acute respiratory distress syndrome (ARDS), possibly leading to the sequential occurrence of chronic lung disease, which is a hallmark comorbidity of post-COVID-19 infection [1]. The absence of innate immunity to COVID-19, a novel virus affecting the human immune system, likely contributes to its rapid spread. Upon COVID-19 infection, the rapid multiplication of the virus in the human body can introduce antigens that activate immune cells [1]. When these immune cells produce excessive amounts of pro-inflammatory molecules known as cytokines including interleukins (IL), TGF-β, and TNF-α, the body may undergo a phenomenon known as cytokine storm [1,2]. This phenomenon is characterized by the increased production of prooxidants, pro-inflammatory reactions, reactive oxygen species, inflammatory eicosanoids, inflammatory chemokines, and cytokines [1,2]. Although a sufficient amount of cytokines is necessary to initiate pathogen clearance, excessive secretion of inflammation-inducing molecules may lead to multi-organ damage [2]. Approximately one-quarter of patients with COVID-19 experience pulmonary function loss, progressing to critical illness requiring intensive care unit (ICU) intubation [3]. Furthermore, these reactions may impair various aspects of adaptive immunity including CD4+ and CD8+ T cell reduction and the decreased functionality of CD4+ T in producing interferon (IFN)-γ [4].

In COVID-19-induced ARDS, nutrition intervention can promote immune function and attenuate inflammatory reactions [5]. A meta-analysis by Ojo et al. found that the administration of enteral nutrition in critically-ill patients with COVID-19 reduced mortality and led to a lower sequential organ failure assessment (SOFA) score [6]. Proper nutrition combined with early enteral nutrition interventions have been proven to improve patient outcomes in hospitalized patients in various pathological settings [7,8,9]. Besides aiding patients in achieving their protein and energy requirements, micronutrients delivered through enteral nutrition can aid in modulating the immune system and improving prognosis [10]. Glutamine and arginine are conditionally essential amino acids that are required in high amounts by critically-ill patients [11,12]. Two studies found positive effects of supplementation with these amino acids in respiratory-related diseases including decreased lipid peroxidation and reduced hospital stays [11,12].

A similar study was performed using an arginine-enriched formula in patients with COVID-19 [12], but the administration of an arginine- and glutamine-enriched formula in patients with COVID-19 has not been documented. Given the desirable outcomes of enteral nutrition and improved patient prognosis through the administration of enhanced enteral formula, this study aimed to assess the effects of immune-enhancing formula enhanced with arginine and glutamine on the clinical signs and biomarkers of patients with severe COVID-19.

## 2. Materials and Methods

This retrospective observational study used data from all patients admitted to the ICU with severe COVID-19. The data were obtained from the Taipei Tzu Chi Hospital COVID-19 Biobank during the COVID-19 pandemic in 2021 (May 2021 to July 2021), which coincided with a sharp increase in the B.1 variant [13], the variant with the highest mortality rate of 4.19% [14]. This study included all patients with samples registered in the biobank, regardless of their age and sex. Patients or legal guardians were fully informed of the collection of blood samples and related information for research purposes. Participants were assured of their right to withdraw from the study at any time. Mild-to-moderate cases of patients with COVID-19 who died within 7 days of hospital admission, patients lacking blood test results within 7 days of hospital admission, or those with concurrent infection within 48 h of hospital admission were excluded, as insufficient data for comparison were available, and additional infections may exacerbate inflammatory reactions and affect the blood test values. Formula selection was determined through consultations between the attending physician and nutritionists.

**Figure 1:**
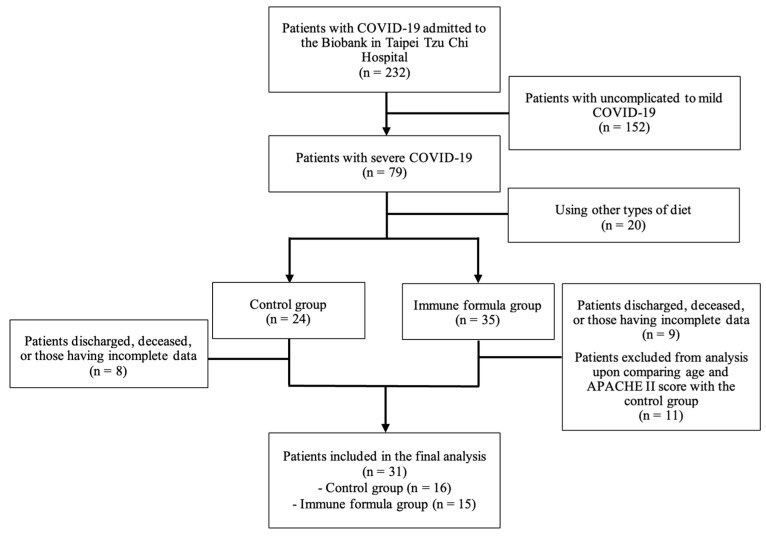
Flow diagram of patient enrollment.

Among the 232 patients in the biobank enrolled in this study, 152 with mild-to-moderate COVID-19 were excluded, leaving 79 patients with severe COVID-19 for recruitment. After excluding 20 patients who used alternative diets, the remaining patients were divided into the control and immune formula groups according to their diet. During the study period, 17 patients were discharged or died. After matching for age, sex, height, weight, and APACHE II score to best match those of the control group, 11 patients from the immune formula group were excluded. Thirty-one patients (16 in the control group and 15 in the immune formula group) with complete data were included in the final analysis (Figure 1).

### 2.1. Immune-Enhancing Formula

Enteral nutrition was administered via nasogastric tubes upon admission to the ICU until discharge or transfer to the general ward. These formulae were divided into the standard and immune-enhancing formulae. The commercially available Efione Nu-Immu powder formula was used in this study (Supplementary Table S1). Compared with the standard formula, the immune-enhancing formula contained arginine and glutamine, with protein accounting for 22% of the total caloric content. The energy requirements of the patients were calculated by a hospital nutritionist. Nutrient and calorie intake calculations were derived from the documented amount of formula consumed as recorded in the ICU medical records.

### 2.2. Data Collection and Analysis Methodology

Demographic data (including age and sex), anthropometric measurements (including weight and height), disease-related metrics (including resting energy expenditure, REE; total energy requirement, TER; and acute physiology and chronic health evaluation II, APACHE II), and duration of hospitalization were obtained from each participant’s medical records. The weight and height of ambulatory patients were measured using a digital scale and stadiometer. If these methods were not possible, a patient bed with a weighing feature and knee height was used to obtain the weight and height, respectively. BMI was calculated using the body weight recorded at ICU admission. REE was calculated using the Harris–Benedict equation, and the resulting value was multiplied by a suitable activity and stress value as assessed by a hospital nutritionist to obtain the TER value. The APACHE scoring was performed by the attending physician immediately after admission and before the patient was discharged from the ICU. Serum and plasma samples were sourced from Taiwan Biobank, where the samples were stored at −80 °C until analysis. The levels of IL-1β, IL-2, IL-4, IL-6, IL-8, IL-10, and IFN-γ were analyzed using the enzyme-linked immunoassay kit from Mybiosource (San Diego, CA, USA). Captured antibodies were added to a 96-well plate and incubated overnight. After washing with buffer, standard solutions and samples were added and allowed to react for two hours at approximately 27 °C. Diluted detection antibodies were added after another washing step. After reacting for one hour at room temperature and washing, 3,3′,5,5′-tetramethylbenzidine reagent was added, and the plate was incubated in darkness for 5–10 min. The reaction was stopped, and the absorbance of the samples was measured at 450 nm using an enzyme-linked immunoassay reader.

### 2.3. Statistical Analysis

Data were analyzed using R statistics software for MacOS (version 4.1.1; R Software for Statistical Computing, Vienna, Austria). Categorical variables are shown as counts (%), while continuous variables are shown as the mean ± standard deviation. Fisher’s exact test, the Wilcoxon rank-sum test, Wilcoxon signed-rank test, and independent sample *t*-test were used to analyze the differences between the data of the two groups. The level of significance was set at *p* < 0.05.

## 3. Results

**Figure 2:**
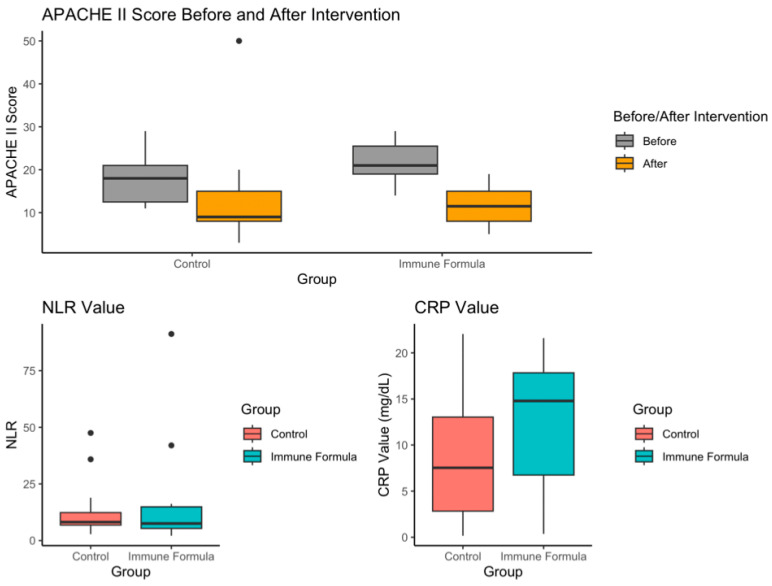
APACHE II score, NLR, and CRP values of the enrolled patients.

Table 1 and Figure 2 present basic information on the participants included in the analysis.

A significant difference was observed in the APACHE II scores between the two groups upon admission to the ICU. The APACHE II score of the immune formula group was significantly higher than the control group (21.7 ± 4.3 vs. 17.6 ± 5.9, *p* = 0.046), but there was no significant difference in the APACHE II score at the time of discharge from the ICU (13.2 ± 12.0 vs. 11.7 ± 5.4, *p* = 0.859). There was a notable increase in body weight after intervention using the immune formula (*p* = 0.016), but this phenomenon was not observed in the control group (*p* = 0.530). No significant differences were observed in sex, age, height, weight, body mass index (BMI), REE, or TER calculated using usual body weight (UBW) or total days of hospitalization between the groups.

**Figure 3:**
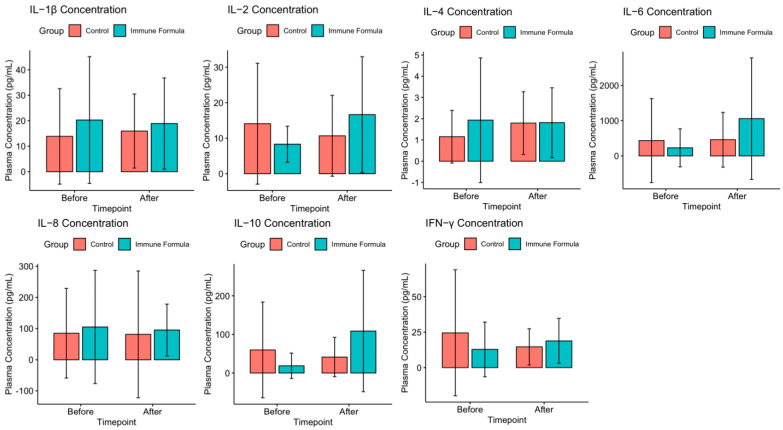
Cytokine level of the enrolled patients pre-and post-intervention.

Analysis of IL-1β, IL-2, IL-4, IL-6, IL-8, IL-10, and IFN-γ at the baseline indicated that the two groups did not differ at the baseline (Figure 3, Supplementary Table S2). After the nutritional intervention, the inflammatory marker values between the two groups did not reach statistical significance (Supplementary Table S2).

When analyzing the intra-group differences, a significant increase was observed in IL-4 within the control group before and after intervention (Figure 3, Supplementary Table S3; 1.15 ± 1.24 vs. 1.79 ± 1.48 pg/mL, *p* = 0.038). Conversely, the IL-6 and IL-10 levels in the immune formula group (Figure 3, Supplementary Table S3) exhibited a significant increase after intervention (IL-6: 228.75 ± 539.99 vs. 1058.33 ± 1725.60 pg/mL; IL-10: 18.99 ± 32.89 vs. 108.76 ± 157.03 pg/mL; *p* = 0.033 and *p* = 0.014, respectively). However, there were no changes for IL-1β, IL-2, IL-8, and IFN-γ in either group.

A comparison of the value change between the baseline and post-intervention values between the two groups revealed a significant difference in IL-10 patterns (Table 2, Supplementary Figure S1; −18.41 ± 129.60 vs. 89.77 ± 161.40 pg/mL, *p* = 0.048).

## 4. Discussion

This study aimed to assess the effects of an immune-enhancing formula compared with a standard formula in patients with severe COVID-19. Our findings revealed that although patients receiving the immune-enhancing formula had a higher APACHE II score, their APACHE II score was reduced to a level similar to the control group after the intervention, indicating a significant improvement in clinical symptoms. The body weight of the intervention group was also significantly higher after the intervention. We speculate that the lower body weight of the intervention group at admission may be correlated with the period of malnutrition of critical patients prior to admission to the ICU [15]. The significant increase in body weight is likely to result from adequate nutrition. Additionally, upon ICU admission, patients who were administered the immune-enhancing formula showed significantly higher IL-10 levels. The neutrophil-to-lymphocyte ratio (NLR) reflects the immune response during infection and is the major predictor of mortality in COVID-19 patients [16,17]. This was not significantly different between groups, signifying that the two groups had similar immune response conditions, although the lack of difference was largely attributed to the great variance within the group. Nonetheless, the NLR value was elevated in the two groups. The CRP values of all the ICU-admitted patients were slightly elevated.

During COVID-19 infection, the body initiates innate and adaptive immune responses, leading to the production of pro-inflammatory cytokines and chemokines [18]. The secretion of these pro-inflammatory substances increases the permeability of the vasculature and disrupts endothelial integrity, possibly leading to ARDS and pulmonary edema, which are features commonly observed in severe COVID-19 [19].

Among the cytokines secreted during viral infection, IL-10 is an important immune regulatory factor, and its role is usually linked to the attenuation of inflammatory conditions. IL-10 can suppress tissue damage via the reduction of a protein that stabilizes cytokine mRNA, known as the HuR protein, promoting a balance between pro-inflammatory and anti-inflammatory responses [20]. Our findings revealed a significant increase in serum IL-10 levels in the immune-enhancing formula group post-intervention, indicating that the formula may affect anti-inflammatory and immune responses in patients with severe COVID-19. It is important to consider how the increase in IL-10 may also be linked to CD8+ T cell proliferation and activation as well as the increase in cytokine production [21]. However, the change could only be observed in IL-10, and no significant changes were observed in other cytokines; this effect requires further investigation.

The increase in serum IL-10 levels may be related to the composition of the immune-enhancing formula used in this study. Previous studies have explored the impact and prognosis of immune-enhancing formulations on various diseases. The oral administration of enteral formulas has been reported to reduce the pro-inflammatory cytokine IL-6 and C-reactive protein levels in patients with severe COVID-19 [22]. The immune-enhancing formula used in our study contained glutamine and arginine, which are quickly metabolized by critically-ill patients [11,12].

The administration of an immune-enhancing formula enriched with arginine and PUFAs in critically-ill patients led to a reduction in vasopressor use and continuous renal replacement therapy [23]. In another study, l-arginine supplementation decreased the length of hospital stay and reduced the need for respiratory support in the short-term [12]. Arginine is involved in the regulation of immune responses during viral infection. During the initial stages of pathogen invasion, arginine is the substrate of nitric oxide production that is involved in the cytotoxic activity of immune cells. To counter the hyperactivation of immune cells after viral eradication, anti-inflammatory macrophages metabolize arginine using the enzyme arginase. Their activity is related to down-tuning the inflammatory processes to prevent organ damage [24]. Arginine has also been found to promote the survival of T cells [25].

Glutamine deficiency has been linked to the development of COVID-19-associated pneumonia [26]. A study that administrated 10 g of glutamine thrice daily found decreased IL-1β, TNF-α, and hs-CRP in COVID-19 outpatients [27]. The mechanistic pathways of how glutamine exhibits its anti-inflammatory properties have been well-documented. Glutamine regulates gene expression related to protein synthesis and cell repair such as heat shock proteins. Furthermore, immune cells including macrophages and leukocytes use glutamine as their primary energy source during viral infection. Aside from being an energy source, the metabolites stimulate the proliferation and expression of immune cell markers [28].

Inflammatory cytokine reduction was not observed in our study. Still, an increase in anti-inflammatory IL-10 levels was observed, which may correlate with a decrease in the overall APACHE II score post-intervention. The current literature regarding the relationship between arginine and IL-10 is still lacking, but glutamine may stimulate the production of IL-10 in regulatory B cells via the mTOR pathway [29]. Although other works in the literature have suggested that these amino acids may reduce other inflammatory cytokines, our findings did not detect such events, which may require future investigation.

Glutamine and arginine are also recognized for their regulatory effects on intestinal barrier function and inflammatory responses [30]. Because critically-ill patients tend to have insufficient intestinal perfusion, inflammation in the intestinal mucosa and increased permeability may occur. Immunonutrition supports barrier repair and function and stimulates intestinal microorganisms to produce short-chain fatty acids (SCFAs), which aid in maintaining the intestinal barrier. Among the SCFAs produced, butyrate regulates the maturation of dendritic cells, increases the number of regulatory T cells and the concentration of anti-inflammatory cytokines, and represses the production of anti-inflammatory cytokines [31]. Although this study did not examine the gut microbiota and its metabolites in the participants, it can be speculated that immunonutrition may inhibit the inflammatory response by regulating intestinal function.

Our findings demonstrate that the use of arginine- and glutamine-enriched enteral formulas improved the inflammatory markers of patients with critical COVID-19. These findings may be applied to patients with severe COVID-19 to improve disease outcomes. However, this study has several limitations. First, this was a retrospective, single-center study with a limited sample size, which increased the risk of selection bias. Second, the nature of the study design and limited access to patient data limited the possibility to account for other confounding factors such as comorbidities. The interpretation of the results should consider the presence of other variables. Future studies should validate the phenomena observed in this study through double-blind, prospective studies.

## Figures and Tables

**Table 1:** Baseline characteristics of patients with COVID-19.

Baseline Characteristics	All Participants (*n* = 31)	Group		*p*-Value
		Control (*n* = 16)	Immune Formula (*n* = 15)	
Sex				1.000
Female	10 (32.3)	5 (31.3)	5 (33.3)	
Male	21 (67.7)	11 (68.8)	10 (66.7)	
Age	64.2 ± 10.9	62.0 ± 10.2	66.5 ± 11.4	0.133
Height (cm)	163.0 ± 7.7	164.2 ± 7.3	161.7 ± 8.2	0.394
Weight on admission (kg)	68.0 ± 11.8	70.1 ± 13.2	65.8 ± 10.1	0.373
Weight at discharge (kg) ^a^	71.0 ± 11.9	70.94 ± 14.1	71.0 ± 9.8	0.648
BMI (kg/m^2^)	25.5 ± 5.3	26.2 ± 6.4	24.8 ± 3.9	0.984
APACHE II ^a^				
Admission to ICU	19.6 ± 5.5	17.6 ± 5.9	21.7 ± 4.3	0.046 *
Discharge from ICU	12.7 ± 10.3	13.2 ± 12.0	11.7 ± 5.4	0.859
WBC (10^3^/µL)	8.56 ± 4.44	8.82 ± 2.68	8.28 ± 5.86	0.118
Lymphocyte count (10^3^/µL)	0.80 ± 0.43	0.84 ± 0.46	0.75 ± 0.40	0.527
NLR	14.22 ± 17.98	12.77 ± 12.16	15.77 ± 23.01	0.489
CRP (mg/dL)	10.26 ± 7.19	8.57 ± 7.05	12.07 ± 7.12	0.173
REE (kcal)	1396.9 ± 252.6	1462.4 ± 287.0	1327.0 ± 195.7	0.109
TER UBW (kcal)	1994.1 ± 390.6	2105.9 ± 413.4	1874.9 ± 338.2	0.101
Total length of stay (days)	33.3 ± 20.4	30.6 ± 15.6	36.1 ± 24.7	0.621

^a^ Some data were unavailable; * *p* < 0.05; Abbreviations: BMI, body mass index; COVID-19, coronavirus disease 2019; ICU, intensive care unit; WBC, white blood cells, NLR; neutrophil-to-lymphocyte ratio; CRP; c-reactive protein; REE, resting energy expenditure; TER UBW, total energy requirement (calculated using) usual body weight.

**Table 2:** Changes in the pre-intervention and post-intervention values in the two groups.

**Biomarkers**	**Groups**		***p*-Value**
	**Control** **(*n* = 16)**	**Immune Formula** **(*n* = 15)**	
IL-1β (pg/mL)	2.06 ± 21.86	−1.37 ± 32.78	0.733
IL-2 (pg/mL)	−3.42 ± 22.77	7.33 ± 16.26	0.144
IL-4 (pg/mL)	0.64 ± 1.91	0.32 ± 1.73	0.632
IL-6 (pg/mL)	23.69 ± 1272.36	829.58 ± 1743.52	0.150
IL-8 (pg/mL)	−3.60 ± 252.80	−9.61 ± 213.78	0.944
IL-10 (pg/mL)	−18.41 ± 129.60	89.77 ± 161.40	0.048 *
IFN-γ (pg/mL)	−9.90 ± 49.71	6.07 ± 20.69	0.258

* *p* < 0.05. Abbreviations: IFN, interferon; IL, interleukin.

## Data Availability

The datasets generated during and/or analyzed during the current study are available from the corresponding author on reasonable request.

## Supplementary Materials

The following supporting information can be downloaded at: https://www.mdpi.com/article/10.3390/biomedicines13020309/s1, Table S1: Nutrient composition of the immune-enhancing formula; Table S2: Intergroup comparison of inflammatory markers pre- and post-intervention; Table S3: Intragroup comparison of inflammatory markers pre- and post-intervention. Figure S1: Cytokine changes of enrolled patients post-intervention.

## Abbreviations

The following abbreviations were used in this manuscript:

COVID-19	Coronavirus disease 2019
ARDS	Acute respiratory distress syndrome
IL	Interleukins
SOFA	Sequential organ failure assessment
BMI	Body mass index
REE	Resting energy expenditure
TER	Total energy requirement
APACHE II	Acute physiology and chronic health evaluation II
UBW	Usual body weight
SCFAs	Short-chain fatty acids
