# Supplementary material for: Proof of Concept: Effects of an Immune-Enhancing Formula on Clinical Markers of Critical Coronavirus Disease 2019 Cases
Source: Biomedicines. 2025 Jan 27;13(2):309. doi: 10.3390/biomedicines13020309 (PMC11852627; doi:10.3390/biomedicines13020309)

biomedicines

Supplementary Material

Supplementary Table S1. Nutrient composition of the immune-enhancing formula

Item	Per 100g
Energy (kcal)	422
Protein (g)	23.2
Fat (g)	12.2
Saturated fatty acids (g)	6.1
Trans fatty acids (g)	0
Medium chain fatty acids (g)	5.6
Carbohydrate (g)	54.9
Sodium (mg)	316
Arginine (g)	4.9
Glutamine (g)	4.7

Abbreviations: kcal, kilocalorie; g, gram; mg, milligram

[1]

biomedicines

Supplementary Table S2. Intergroup comparison of inflammatory markers pre- and post-intervention

Biomarkers	Pre-intervention		p-value	Post-intervention		p-value
	Control (n = 16)	Immune formula (n = 15)		Control (n = 16)	Immune formula (n = 15)	
IL-1β (pg/mL)	13.88 ± 18.77	20.27 ± 24.89	.313	15.94 ± 14.52	18.90 ± 17.91	.890
IL-2 (pg/mL)	14.09 ± 17.02	8.56 ± 4.98	1.000	10.67 ± 11.38	15.89 ± 15.99	.573
IL-4 (pg/mL)	1.15 ± 1.24	1.26 ± 1.18	.635	1.79 ± 1.48	1.58 ± 1.40	.607
IL-6 (pg/mL)	434.15 ± 1189.70	228.75 ± 539.99	.309	457.84 ± 776.02	1058.32 ± 1725.60	.213
IL-8 (pg/mL)	84.84 ± 143.98	104.95 ± 181.66	.567	81.24 ± 203.53	95.33 ± 83.10	.060
IL-10 (pg/mL)	59.95 ± 123.97	18.99 ± 32.89	.238	41.54 ± 51.00	108.76 ± 157.03	.009
IFN-γ(pg/mL)	24.52 ± 44.48	12.82 ± 19.31	.191	14.63 ± 12.82	18.89 ± 15.86	.417

Values calculated using the Wilcoxon rank-sum test

*p < .05, **p < .01, ***p < .001

Supplementary Table S3. Intragroup comparison of inflammatory markers pre- and post-intervention

Biomarkers	Control		*p*-value	Immune formula		*p*-value
	Pre-intervention	Post-intervention		Pre-intervention	Post-intervention	
IL-1β (pg/mL)	13.88 ± 18.77	15.94 ± 14.52	.597	20.27 ± 24.89	18.90 ± 17.91	.842
IL-2 (pg/mL)	14.09 ± 17.02	10.67 ± 11.38	.760	8.56 ± 4.98	15.89 ± 15.99	.109
IL-4 (pg/mL)	1.15 ± 1.24	1.79 ± 1.48	.038*	1.26 ± 1.18	1.58 ± 1.40	.293
IL-6 (pg/mL)	434.15 ± 1189.70	457.84 ± 776.02	.313	228.75 ± 539.99	1058.33 ± 1725.60	.033*
IL-8 (pg/mL)	84.84 ± 143.98	81.24 ± 203.53	.266	104.95 ± 181.66	95.33 ± 83.10	.670
IL-10 (pg/mL)	59.95 ± 123.97	41.54 ± 51.00	.660	18.99 ± 32.89	108.76 ± 157.03	.014*
IFN-γ(pg/mL)	24.52 ± 44.48	14.63 ± 12.82	.798	12.82 ± 19.31	18.89 ± 15.86	.330

Values calculated using the Wilcoxon signed rank test

$^*p < .05$, $^{**}p < .01$, $^{***}p < .001$

biomedicines

Supplementary Figure S1. Cytokine changes of enrolled patients post-intervention

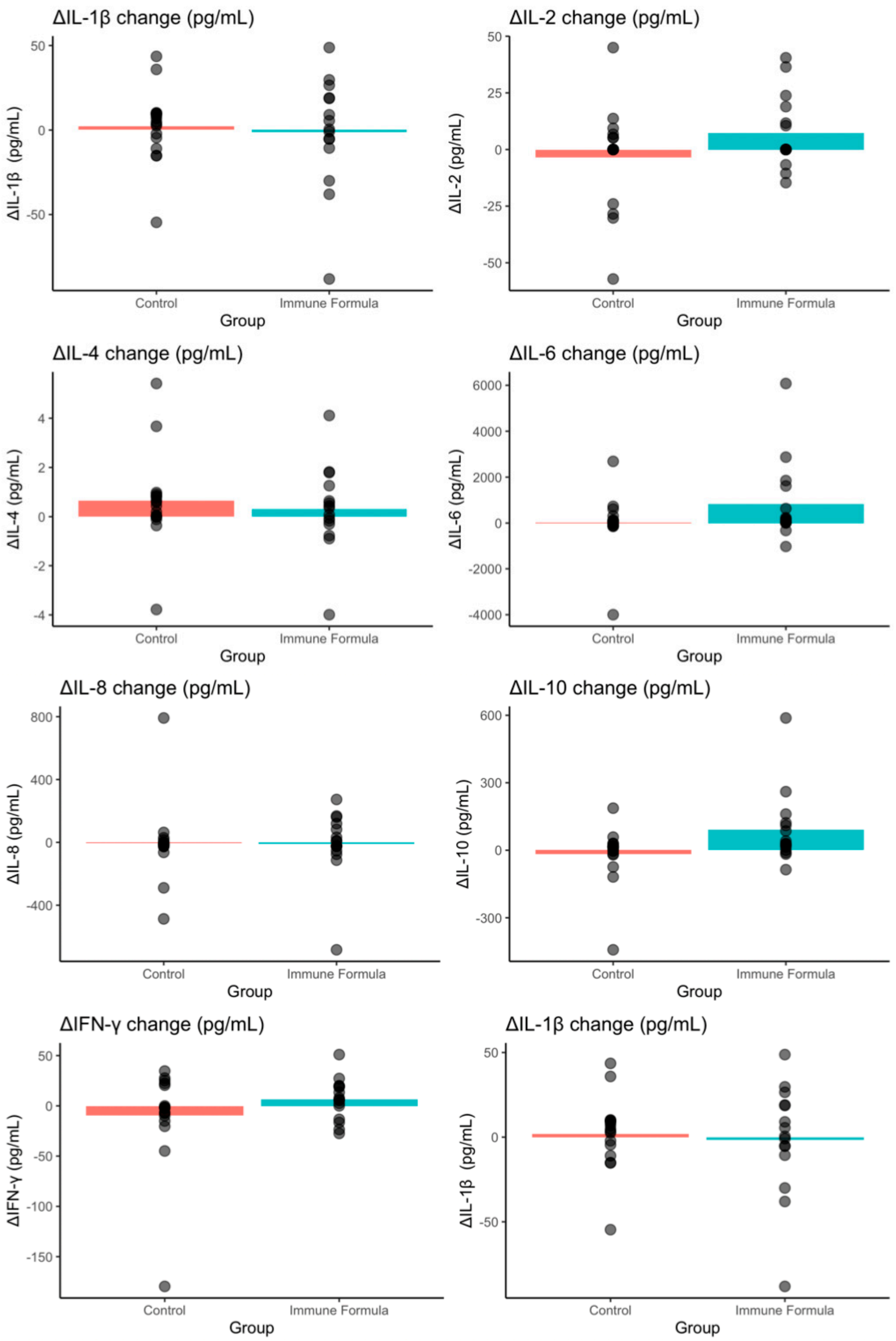

Supplement: Supplementary file 1 [file biomedicines-13-00309-s001.zip › biomedicines-3435904-supplementary.pdf]
